# Age‐dependent impairment of the erythropoietin response to reduced central venous pressure in HFpEF patients

**DOI:** 10.14814/phy2.14021

**Published:** 2019-02-28

**Authors:** David Montero, Thomas Haider, Jens Barthelmes, Jens P. Goetze, Silviya Cantatore, Carsten Lundby, Isabella Sudano, Frank Ruschitzka, Andreas J. Flammer

**Affiliations:** ^1^ University Heart Center University Hospital Zurich Zurich Switzerland; ^2^ Libin Cardiovascular Institute of Alberta, Faculty of Kinesiology University of Calgary Calgary Canada; ^3^ Institute of Physiology University of Zurich Zurich Switzerland; ^4^ Department of Clinical Biochemistry Rigshospitalet University of Copenhagen Copenhagen Denmark; ^5^ Department of Clinical Medicine Rigshospitalet University of Copenhagen Copenhagen Denmark

**Keywords:** Central venous pressure, erythropoietin, heart failure, orthostatic tolerance

## Abstract

Despite growing research interest in the pathophysiology of heart failure with preserved ejection fraction (HFpEF), it remains unknown whether central hemodynamic alterations inherently present in this condition do affect blood pressure and blood volume (BV) regulation. The present study sought to determine hemodynamic and endocrine responses to prolonged orthostatic stress in HFpEF patients. Central venous pressure (CVP) assessed via the internal jugular vein (IJV) aspect ratio with ultrasonography, arterial pressure and heart rate were determined at supine rest and during 2 hours of moderate (25–30°) head‐up tilt (HUT) in 18 stable HFpEF patients (71.2 ± 7.3 years), 14 elderly (EC), and 10 young (YC) healthy controls. Parallel endocrine measurements comprised main BV‐regulating hormones: pro‐atrial natriuretic peptide, copeptin, aldosterone, and erythropoietin (EPO). At supine rest, the IJV aspect ratio was higher (>30%) in HFpEF patients compared with EC and YC, while mean arterial pressure was elevated in HFpEF patients (98.0 ± 13.1 mm Hg) and EC (95.6 ± 8.3 mm Hg) versus YC (87.3 ± 5.0 mm Hg) (*P *<* *0.05). HUT increased heart rate (+10%) and reduced the IJV aspect ratio (−52%), with similar hemodynamic effects in all groups (*P* for interaction ≥ 0.322). The analysis of endocrine responses to HUT revealed a group×time interaction for circulating EPO, which was increased in YC (+10%) but remained unaltered in HFpEF patients and EC. The EPO response to a given reduction in CVP is similarly impaired in HFpEF patients and elderly controls, suggesting an age‐dependent dissociation of EPO production from hemodynamic regulation in the HFpEF condition.

## Introduction

The condition of heart failure with preserved ejection fraction (HFpEF) entails prominent hemodynamic alterations challenging the pressure gradient driving venous return as well as the regulation of blood volume (BV) (Miller 2016; Montero et al. 2017). A fairly constant BV in healthy humans depends on stretch receptors located predominantly in the atria and veno‐atrial junctions where they sense central venous pressure (CVP), which reflects the filling state of the cardiovascular system (Guyton 1957; Gauer and Henry 1963). BV‐regulating hormones are released in response to changes in CVP via autonomic pathways (Segar and Moore 1968; Egan et al. 1984; Bie et al. 1986; Sander‐Jensen et al. 1986; Montero et al. 2016a). These hormones control the two major constituents of BV, that is, plasma volume (PV) and red blood cell volume (RBCV) (Montero et al. 2016a). A reduction in CVP induced by either moderate blood loss or head‐up tilt (HUT) activates the renin–angiotensin–aldosterone system (RAAS) and enhances the production of vasopressin and erythropoietin (EPO) (Ehmke et al. 1995; Roberts et al. 2000; Montero et al. 2016a), the principal hormones regulating fluid homeostasis and erythropoiesis, respectively. On the other hand, infusion of BV expanders or sustained head‐down tilt leading to increased CVP prompts the release of natriuretic peptides by atrial cells in parallel to decreasing circulating EPO levels (Gunga et al. 1996; Breymann et al. 2000) eventually resulting in substantial reductions in PV and RBCV (Fortney et al. 1994). Therefore, in the presence of a chronic increase in CVP and possibly altered stretch receptor sensitivity due to cardiovascular stiffening, as is indeed characteristic of HFpEF patients (Guazzi et al. 2011; Reddy and Borlaug 2016; Redfield 2016), the regulation of BV could be compromised. In this regard, recent investigations underline the clinical relevance of fluid dysregulation and the need to reassess the complex pathophysiology of congestion in the HFpEF condition (Miller 2016; Miller and Mullan 2016; Montero et al. 2017). The impairment of central pressure/volume sensors could primarily contribute to endocrine‐related specific alterations in PV as well as RBCV in HFpEF patients (Miller 2016; Montero et al. 2016b; Montero et al. 2017). This would concur with the high prevalence of true anemia (RBCV deficit) in HFpEF cohorts along with the inverse linear relationship between BV and ejection fraction in the heart failure population (Montero et al. 2017). Yet, relatively little is currently known regarding the hemodynamic and endocrine regulation of intravascular volume in the HFpEF condition. Integrative experimental research designed to pinpoint hemodynamic and endocrine responses to changes in CVP may provide sound evidence, but to our knowledge, this remains to be materialized.

The present study experimentally assessed for the first time whether central hemodynamic alterations inherently present in the HFpEF condition alter blood pressure and BV regulation during an orthostatic challenge. Specifically, the purpose of this study was to assess the impact of prolonged moderate HUT on central hemodynamics and major BV‐regulating hormones in stable HFpEF patients and healthy controls including age‐matched elderly (EC) and young (YC) individuals. We hypothesized that hemodynamic and endocrine responses to reduced CVP will be impaired in HFpEF patients compared with control individuals, independently of the age status.

## Methods

### Ethical approval

The study was approved by the Ethical Commission of Zurich (BASEC‐Nr. 2016‐02167, KEK‐ZH‐Nr. 2015‐0044) and conducted in accordance with the declaration of Helsinki. Prior to the start of the experiments, informed oral and written consents were obtained from all participants.

### Study population

Stable HFpEF patients (left ventricular ejection fraction (LVEF) = 55.7 ± 4.9%) were recruited from the HF outpatient clinic of the University Hospital of Zurich. Inclusion criteria comprised: signs and symptoms of HF (Ponikowski et al. 2016), LVEF > 50%, elevated levels of N‐terminal pro b‐type natriuretic peptide (NT‐proBNP > 125 pg·mL^−1^), relevant structural/functional cardiac alterations, and no history of iron‐deficiency anemia. Healthy age‐ and sex‐matched individuals (EC) were recruited from the community and excluded if they presented any chronic medical illness, were taking daily prescription medications, had current medical symptoms, or were performing aerobic exercise on a regular basis. An additional group of healthy young control individuals (YC) enrolled in a parallel investigation (Montero et al. 2016a) were included in order to determine the influence of age.

### Experimental design

The tilt‐table test was performed after fasting overnight in a quiet room with controlled temperature between 22 and 24°C. Patient's regular medication remained unaltered before study assessments. Following 15 min of supine rest, patients were tilted to 25–30° (i.e., HUT) for 120 min. A climbing harness (bicycle saddle for YC) gently held the body weight of individuals at the hip level throughout the HUT protocol. Hemodynamic variables and blood samples were obtained at supine rest and discrete time points (20, 60, and 120 min) during HUT. Blood samples at the last time point (120 min) were not available in YC due to specific protocol characteristics. All blood samples were centrifuged and stored at −80°C until analyzed.

### Experimental measures

#### Hemodynamics

The internal jugular vein (IJV) aspect ratio, a surrogate marker of central venous pressure (CVP), was determined at the level of the cricoid cartilage using the method described by Keller et al. (2009). In brief, the left IJV was assessed by means of a 7‐MHz linear array probe attached to a high‐resolution ultrasound device (SonixTouch, BK Ultrasound, USA). After obtaining an optimized IJV image, a 20‐s B‐mode cine loop was obtained and reviewed frame by frame to identify the largest cross‐sectional area (during expiration), and vessel dimensions were recorded. The IJV height was divided by its width to obtain the aspect ratio. Systemic arterial pressure was measured on the arm with an automated system (Microlife BP3AC1‐1PC, Omron, Switzerland). In addition, pulse wave analysis (PWA) was performed on radial artery pressure waveforms (SphygmoCor, AtCor Medical, Australia) and carotid artery distensibility was determined by means of high‐resolution ultrasound (SonixTouch, BK Ultrasound, USA) (Van Bortel et al. 2002). The distensibility coefficient (DC) was calculated according to the following formula:

$$\text{DC}=\left(2\Delta D\times D+\Delta D^{2}\right)/\left(\text{PP}\times D^{2}\right)$$

where *D* is arterial diameter, ∆*D* is distension, and PP is pulse pressure. DC represents the reciprocal value of arterial stiffness.

#### Laboratory parameters

Blood samples (5 mL) from the antecubital vein were collected anaerobically in heparinized glass syringes. Serum creatinine was measured via the kinetic Jaffe reaction (Hitachi P‐Modular system, Roche Diagnostics, Switzerland) and glomerular filtration rate (eGFR) was calculated by the CKD‐EPI Creatinine Equation (2009) according to guidelines from the National Kidney Foundation (Inker et al. 2014). Hormones measured in plasma included pro‐ANP, N‐terminal pro‐b‐type natriuretic peptide (NT‐proBNP), aldosterone, erythropoietin, and copeptin (Morgenthaler 2010; Balanescu et al. 2011; Roussel et al. 2014), which is the carboxy‐terminal portion of the precursor of vasopressin used as a surrogate clinical marker owing to its greater stability and reliability (Morgenthaler et al. 2006, 2008). Pro‐atrial natriuretic peptide (pro‐ANP) was assessed with a midregional assay on a Kryptor Plus platform (Thermo‐Fisher, Germany) (Hunter et al. 2011), while NT‐proBNP was determined by immunoassay (Elecsys NT‐proBNP, Roche Diagnostics, Switzerland). Copeptin was assessed by means of an automated immunofluorescent assay (Thermo Fisher Scientific BRAHMS, Germany) (Balanescu et al. 2011; Roussel et al. 2014). A competitive enzyme immunoassay (R&D Systems Inc., USA) was used to determine aldosterone. EPO was measured via the Human EPO Quantikine IVD ELISA Kit (R&D Systems Inc., USA). Furthermore, plasma albumin levels were assessed with ALB reagent in conjunction with UniCel^®^ DxC 600/800 and Synchron^®^ Systems Multi Calibrator (Beckman Coulter, USA).

### Statistical analysis

Statistical analysis was performed using SPSS 22.0 (SPSS, Chicago, IL). Data were tested for normal distribution with the Kolmogorov–Smirnov test, homogeneity of variances with Levene's test, and sphericity with Mauchly's test. Data not‐normally distributed, violating the assumptions of homogeneity of variances and/or sphericity were logarithmically transformed before parametric testing. Fisher's exact test and one‐way ANOVA with Tukey post hoc tests were used to compare baseline variables in HFpEF patients and control individuals. Tilt‐table experiments were analyzed with two‐way repeated measures ANOVA with “time” and “group” as within‐ and between‐subject factors, respectively, along with the interaction among these factors. Pairwise comparisons were performed using Student's paired *t* test. Data are reported as mean ± SD unless otherwise stated. A two‐tailed *P*‐value less than 0.05 (0.10 for interaction) (Durand 2013) was considered significant.

## Results

### Baseline characteristics

Table 1 presents anthropometrical and clinical characteristics of HFpEF patients and control individuals. Body mass index was elevated (*P *<* *0.05) in HFpEF patients compared with EC and YC. Sex distribution was similar among groups. Kidney function, determined by eGFR, was mildly reduced (*P *<* *0.05) in HFpEF patients relative to EC (data not available in YC). Comorbidities comprising hypertension (78%), coronary artery disease (44%), and diabetes mellitus (28%) were the most prevalent among HFpEF patients. With respect to medication, beta‐blockers (67%), loop diuretics (61%), and angiotensin‐converting‐enzyme inhibitors/angiotensin II‐receptor blockers (61%) were common and similarly prevalent.

**Table 1:** Baseline characteristics of HFpEF patients and controls

	HFpEF	EC	YC
*n*	18	14	10
Age (years)	71.2 ± 7.3	70.6 ± 5.5	25.5 ± 2.1^1^^,^ 2
Sex (female/male)	5/13	2/12	0/10
Body mass index (kg·m^−2^)	28.9 ± 4.5	24.8 ± 3.3^1^	22.7 ± 1.3^1^
Body surface area (m^2^)	1.94 ± 0.24	1.91 ± 0.18	1.95 ± 0.09
NT‐proBNP (ng·L^−1^)	859 ± 758	81 ± 43^1^	―
eGFR (mL·min^−1^)	64.6 ± 20.9	82.6 ± 9.8^1^	―
Hct (%)	40 ± 5	44 ± 2^1^	―
Ferritin (*μ*g·L^−1^)	149 ± 103	195 ± 115	―
Albumin (g·L^−1^)	40.0 ± 3.7	41.3 ± 1.8	41.1 ± 2.2
Smoking (yes/no)	3/18	0/15	0/10
Cardiac alterations (%)			
LVH	28	0^1^	0^1^
LAE	78	0^1^	0^1^
Diastolic dysfunction	83	0^1^	0^1^
Atrial fibrillation	28	0^1^	0^1^
Comorbidities (%)			
CAD	44	0^1^	0^1^
HTN	78	0^1^	0^1^
DM	28	0^1^	0^1^
Medication (%)			
ACEI/ARB	61	0^1^	0^1^
BB	67	0^1^	0^1^
Loop DIU	61	0^1^	0^1^
Metformin	11	0^1^	0^1^
Statins	56	0^1^	0^1^

Data are presented as mean ± SD, ratio or %. ACEI/ARB, angiotensin‐converting‐enzyme inhibitors or angiotensin II‐receptor blockers; BB, beta‐blockers; CAD, coronary artery disease; DM, diabetes mellitus; EC, elderly controls; eGFR, estimated glomerular filtration rate; Hct, hematocrit; HFpEF, heart failure with preserved ejection fraction; HTN, hypertension; LAE, left atrial enlargement; Loop DIU, loop diuretics; LVH, left ventricular hypertrophy; NT‐proBNP, N‐terminal pro‐b‐type natriuretic peptide; YC, young controls; —, not available.

1 Significantly different (*P *<* *0.05) compared with HFpEF.

2 Significantly different (*P *<* *0.05) compared with EC.

Hemodynamic and endocrine variables at supine rest are reported in Table 2. The IJV aspect ratio was elevated (*P *<* *0.05) in HFpEF patients compared with EC and YC, while mean arterial pressure was augmented (*P *<* *0.05) in HFpEF patients and EC versus YC. No difference was detected for aortic augmentation index and carotid artery distensibility between HFpEF and EC (data not available in YC). With respect to endocrine variables, all BV‐regulating hormones were elevated in HFpEF patients compared with EC and/or YC.

**Table 2:** Hemodynamic and endocrine variables at supine rest in HFpEF patients and controls

	HFpEF	EC	YC
Central hemodynamics			
IJV aspect ratio	0.71 ± 0.18	0.54 ± 0.21^1^	0.51 ± 0.12^1^
SBP (mm Hg)	138.2 ± 22.7	135.6 ± 15.9	122.0 ± 7.3
PP (mm Hg)	60.2 ± 18.7	59.8 ± 18.4	59.5 ± 3.5
HR (bpm)	61.2 ± 12.3	56.9 ± 8.2	56.4 ± 19.1
Aortic Aix@75 (%)	26.1 ± 9.3	24.8 ± 6.1	―
Carotid distensibility (kPa^−1^·10^3^)	1.6 ± 0.7	2.1 ± 1.2	―
BV‐regulating hormones			
proANP (pmol·L^−1^)	274.8 ± 185.8	133.7 ± 41.0^1^	39.6 ± 12.2^1^
Copeptin (pmol·L^−1^)	18.4 ± 17.9	9.1 ± 12.8	4.8 ± 0.9^1^
Aldosterone (ng·dL^−1^)	106.4 ± 53.3	59.0 ± 18.2^1^	―
EPO (U·L^−1^)	16.2 ± 15.1	7.2 ± 1.8^1^	9.9 ± 2.4

Data are presented as mean ± SD. Aix@75, augmentation index adjusted by heart rate of 75 bpm; EC, elderly controls; EPO, erythropoietin; HFpEF, heart failure with preserved ejection fraction; HR, heart rate; IJV, internal jugular vein; PP, pulse pressure; proANP, pro‐atrial natriuretic peptide; SBP, systolic blood pressure; YC, young controls; —, not available.

1 Significantly different (*P *<* *0.05) compared with HFpEF.

### Hemodynamic responses to head‐up tilt (HUT)

**Figure 1:**
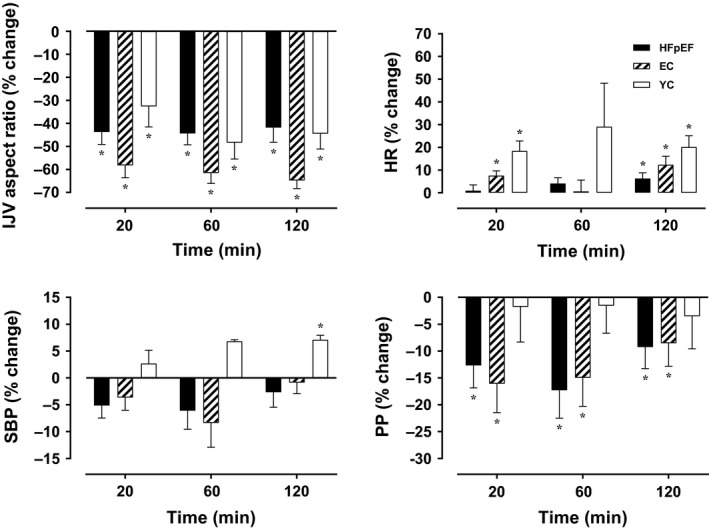
Hemodynamic responses to HUT in HFpEF patients and control individuals. Data are presented as mean ± SEM. and control individuals.^*^ Significant change (*P *<* *0.05) compared with baseline (supine). EC, elderly controls; HFpEF, heart failure with preserved ejection fraction; HR, heart rate; HUT, head‐up tilt; IJV, internal jugular vein; PP, pulse pressure; SBP, systolic blood pressure; YC, young controls.

Figure 1 displays the effects of HUT on hemodynamic variables in HFpEF patients and control individuals. As expected, IJV aspect ratio was decreased throughout HUT (*P* for time < 0.001), with a similar decline observed in all groups (*P* for interaction = 0.322). No definite pattern was detected for systolic blood pressure (SBP) (*P* for time = 0.369, *P* for interaction = 0.457), whereas PP was reduced with HUT (*P* for time = 0.020, *P* for interaction = 0.680). Conversely, heart rate was increased during HUT (*P* for time = 0.045, *P* for interaction = 0.626). None of the hemodynamic effects induced by HUT differed among groups. No sign/symptom of orthostatic hypotension was observed in any individual.

### Endocrine responses to head‐up tilt (HUT)

**Figure 2:**
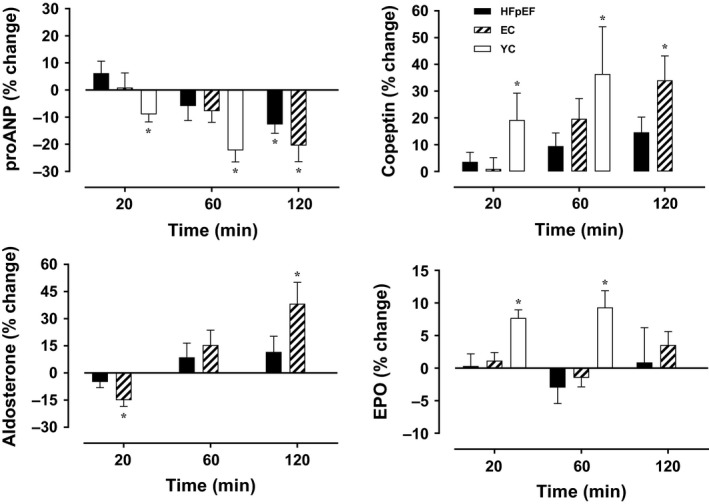
Endocrine responses to HUT in HFpEF patients and control individuals. Data are presented as mean ± SEM. and control individuals. Data was not available in YC for aldosterone and time “120”.^*^ Significant change (*P *<* *0.05) compared with baseline (supine). EC, elderly controls; EPO, erythropoietin; HFpEF, heart failure with preserved ejection fraction; HUT, head‐up tilt; proANP, pro‐atrial natriuretic peptide; YC, young controls.

The effects of HUT on BV‐regulating hormones in HFpEF patients and control individuals are presented in Figure 2. In keeping with a typical response to reduced CVP, proANP was decreased with HUT in all groups (*P* for time < 0.001, *P* for interaction = 0.908). Likewise, HUT increased copeptin (*P* for time = 0.015, *P* for interaction = 0.657) and aldosterone (*P* for time = 0.003 *P* for interaction = 0.520) (data not available in YC). A group × time interaction was detected for EPO (*P* for interaction = 0.061) in that EPO was only increased with HUT in YC. No change in EPO during HUT was noted in HFpEF patients and EC.

## Discussion

This study assessed hemodynamic and endocrine responses to HUT in stable HFpEF patients and healthy controls comprising age‐matched elderly (EC) and young (YC) individuals. The main findings are: (1) HUT elicits similar reductions in IJV aspect ratio and compensatory increases in heart rate in HFpEF patients and control individuals, irrespective of the age status; (2) circulating EPO augments with HUT in YC but remains unaltered in HFpEF patients and EC.

Since the inception of current concepts in cardiovascular physiology in the mid‐20th century, the regulation of the cardiovascular system has been intrinsically linked to its internal volume, that is, BV (Guyton 1957; Gauer and Henry 1963). A clear‐cut example of this relationship is evidenced by the ineludible impact of hemorrhage or BV expansion on CVP and cardiac preload, key hemodynamic variables determining stroke volume via the Frank–Starling mechanism (Guyton and Hall 2011). In turn, volumetrically induced changes in stroke volume are counterbalanced with opposite changes in heart rate and vascular resistance mediated by the autonomic nervous system in order to maintain a steady cardiac output and blood pressure (Robinson et al. 1966; Bonne et al. 2014). Cardiovascular function is thus fundamentally regulated by BV. Reciprocally, BV depends upon intact cardiovascular stretch receptors, also known as baroreceptors, signaling the filling state of the low‐pressure system (veins, right heart, pulmonary circulation, left atrium; comprising approximately 80% of BV) and high‐pressure system (left ventricle, arteries) to the medullary cardiovascular center (Segar and Moore 1968; Egan et al. 1984; Bie et al. 1986; Sander‐Jensen et al. 1986; Montero et al. 2016a). In HFpEF patients, baroreceptor dysfunction and abnormal BV are expected as a result of chronically elevated CVP levels and reduced cardiovascular distensibility (Guazzi et al. 2011; Reddy and Borlaug 2016; Montero et al. 2017). These central phenotypic alterations may hinder the intertwined regulation of blood pressure and volume, including PV and RBCV components, which is a topic being reexamined in light of new experimental evidence in the heart failure field (Hage et al. 2015; Miller 2016; Miller and Mullan 2016; Montero et al. 2016b; Montero et al. 2017). Among the uncertainties, it is currently unknown whether basic compensatory hemodynamic responses to altered BV distribution are impaired in this condition. Our findings indicate that central hemodynamic effects induced by 2 h of HUT, which led to an estimated ~3 mmHg decrease in CVP (Prekker et al. 2013) equivalent to 10% BV loss and 50% reduction in baroreceptor firing rate (Gauer and Henry 1963), and do not differ between HFpEF patients and healthy control individuals (EC, YC). Likewise, no sign or symptom of orthostatic hypotension was detected in HFpEF patients, despite they presented with a 24% increment of baseline IJV aspect ratio (a proxy for CVP (Keller et al. 2009; Montero et al. 2016a)) but normal arterial blood pressure compared with EC. Therefore, the present study demonstrates that HFpEF patients may retain the control of hemodynamic stability against sustained CVP elevation and acute CVP curtailment with HUT, presumably underpinned by a resetting of low‐pressure baroreceptors to operate at higher pressure levels.

Beyond swift hemodynamic responses driven by neural reflexes, endocrine responses are capital to cope with sustained orthostatic challenges. As aforementioned, afferent signals from central baroreceptors regulate the production of BV‐regulating hormones, thereby modulating kidney function according to the “fullness” of the intrathoracic compartment (Segar and Moore 1968; Egan et al. 1984; Bie et al. 1986; Sander‐Jensen et al. 1986; Montero et al. 2016a). When gravity propels BV toward the lower extremities, central baroreceptors are partially unloaded and circulating levels of key hormones governing PV and RBCV such as pro‐ANP, vasopressin, the RAAS cascade, and EPO, are altered in the direction of fluid retention and enhanced erythropoiesis (Montero et al. 2016a). Subsequent volumetric adaptations are essential to avoid the circulatory collapse that would eventually ensue if only cardiovascular adjustments were recruitable in the attempt to preserve cardiac filling (Guyton 1957; Gauer and Henry 1963). Consistent with this rationale, in this investigation HUT prompted changes in circulating proANP (−13 to −22%), copeptin (+13 to +35%), and aldosterone (+9 to +32%), collectively facilitating PV expansion. No statistical difference was detected among groups. In contrast, HUT did not alter circulating EPO in HFpEF patients and EC, differing from the rise in EPO observed in YC. Whilst speculative, the age‐related impairment of the hemodynamic regulation of EPO could contribute to the prevalent RBCV deficit in the HFpEF population (Montero et al. 2017), given that the prominent stimulus for EPO synthesis, that is, the hypoxic drive, effectively operates at very low hematocrit levels (Kurtz and Eckardt 1990; Le Hir et al. 1991; Roberts et al. 2000; Wenger and Kurtz 2011). In fact, provided that pulmonary gas exchange is not impaired, EPO production cannot be spurred by hypoxia‐dependent mechanisms unless RBCV and hemoglobin mass are markedly reduced, particularly when arterial oxygen content is preserved due to a concomitant reduction of PV as observed in heart failure patients long‐term treated with loop diuretics (Anand et al. 1989; Feigenbaum et al. 2000; Bonfils et al. 2010), a common prescription in our study population (Montero and Flammer 2017). Furthermore, it should be noted that baseline EPO levels were elevated in HFpEF patients and presented higher variability compared with healthy control groups. This concurs with previous observations in HF patients showing increased plasma EPO in direct relationship with disease severity (Volpe et al. 1994; George et al. 2005). EPO levels in HF are also a function of hemoglobin concentration, age, sex, and inflammation (Montero et al. 2018), underpinning the complex pathophysiology of HF. Indeed, increased baseline EPO may have limited the responsiveness of EPO‐producing cells to HUT‐related stimuli in the present study. Ultimately, the hemodynamic regulation of EPO production in HFpEF patients may be inadequate to cope with chronic heart failure‐related limitations to the erythropoietic process, for example, hematopoietic bone marrow dysfunction and secondary EPO resistance (Westenbrink et al. 2010; Okonko et al. 2013).

The mechanisms underlying the absent EPO response to reduced CVP in HFpEF and EC are uncertain. Aging is inexorably associated with a progressive stiffening of the circulatory system (Monahan et al. 2001) leading to blunted baroreceptor firing rate attributed to restricted changes in vessel circumference (Kingwell et al. 1995). In consequence, specific baroreflex‐endocrine signaling pathways that stimulate erythropoiesis may be altered with advanced age. In this respect, vasopressin responses, primarily dependent on high‐pressure (aortic and carotid) baroreceptors (Norsk et al. 1993; Thrasher and Keil 1998), are decreased during orthostatic stress in elderly individuals (Rowe et al. 1982). Vasopressin, in parallel to controlling water reabsorption, directly prompt EPO secretion through the activation of V1a receptors(Engel and Pagel 1995) expressed in the renal cortex and medulla (Gozdz et al. 2002; Koshimizu et al. 2006). In healthy young individuals, the increase in circulating EPO induced by moderate HUT is strongly associated with the concomitant increase in copeptin (Montero et al. 2016a). In agreement with prior evidence in elderly individuals (Rowe et al. 1982), the effect of HUT on copeptin was seemingly attenuated and delayed in HFpEF patients and EC. This could be partly attributed to the similarly impaired central arterial distensibility in HFpEF patients and EC (Montero et al. 2016b). Similar to the EPO response, the effect of HUT on copeptin in EC and HFpEF patients could also be influenced by elevated copeptin basal levels, which as a matter of fact are strongly associated with renal dysfunction and poor prognosis in HFpEF patients (Hage et al. 2015). Furthermore, vasopressin release might be inhibited with augmented mean arterial pressure (Gabrielsen et al. 2000), as observed herein in the elderly groups. Further experimental research is needed to establish whether baroreflex‐endocrine mechanisms may explain the age‐dependent impairment of the EPO response to HUT in the HFpEF condition.

### Limitations

First, findings arose from a moderate sample size of stable HFpEF patients, mainly males, thus our conclusions should be taken with caution. Likewise, control individuals did not present comorbidities or risk factors. Therefore, a clear separation between HFpEF and additional conditions cannot be ascertained. Larger prospective studies might buttress the present findings and contrast different HFpEF phenotypes, comorbidities, risk factors, and pharmacotherapies (Montero and Flammer 2017). Second, kidney partial pressure of oxygen (*P*_O2_), a variable regulating EPO production independently of central hemodynamics, was not assessed. Nonetheless, the main determinant of kidney *P*_O2_, that is, glomerular filtration rate, was not altered during HUT in HFpEF patients and control individuals. Third, smoking may have influenced EPO levels (Eisenga et al. 2018) in the 3 HFpEF patients who were smokers. The exclusion of these patients did not affect the results of the study. Finally, we did not include a control intervention for time since previous studies indicate (i) a prevailing impact of HUT over the circadian rhythm of BV‐regulating hormones (Montero et al. 2016a), and (ii) minimal or no diurnal fluctuation of circulating EPO (Gunga et al. 1996; Roberts and Smith 1996).

## Conclusion

The current study demonstrates that prolonged HUT elicits comparable central hemodynamic responses in HFpEF patients and control individuals. In contrast, endocrine responses to reduced CVP comprising increases in circulating EPO are absent in HFpEF patients and age‐matched elderly individuals, suggesting that the hemodynamic regulation of EPO production is age‐dependent. The pathophysiological mechanisms underlying these findings are speculative at present and will have to be characterized in future studies.

## Conflict of Interest

None declared.
